# Paper-based evaporation concentrators: Comparison of linear and radial geometries

**DOI:** 10.1063/5.0129510

**Published:** 2023-01-04

**Authors:** Richard R. A. Syms, Steven Wright

**Affiliations:** EEE Department, Imperial College London, Exhibition Road, London SW7 2AZ, United Kingdom

## Abstract

Paper-based evaporation concentrators with linear and radial geometries are compared. A new method of finding approximate analytic solutions of the advection–dispersion equation is proposed, based on the behavior of concentrators with infinite sources. Analytic approximations are compared with numerical solutions, and the advantage of radial concentration is highlighted: linear concentration rates scale with the square root of the Péclet number Pe while radial rates scale with Pe itself, leading to faster radial concentration beyond a critical value. Experiments are performed with Brilliant Blue FCF dye, using optical transmission and the Beer–Lambert law for quantitation. Dye concentrations are chosen for operation in the linear absorbance regime. Radial concentration is demonstrated under ambient conditions on filter paper disks with 60 mm diameter evaporation areas fed from a perimeter source, in a reverse of the well-known “coffee stain” experiment. Airflow enhanced concentration in strips and wedges is compared directly, using laser-patterned chromatography paper. The advantage of radial concentration is confirmed (and enhanced by diversion of concentrate to the corners of strips) and concentration factors greater than ∼500 (the dynamic range of measurement) are obtained in ∼2 h using 30 mm long columns.

## I. INTRODUCTION

Originally developed as spot tests for common conditions and more recently proposed to improve healthcare in the developing world, lab-on-paper^1^ has emerged as an essential diagnostic tool during the COVID-19 pandemic.^2^ Development has been rapid.^3^ Flow channels have been defined by boundary shaping and printing;^4–6^ programmable delays have been developed to control flow or increase analyte–antibody binding times;^7^ and detection has been achieved using optical transmission,^8^ colorimetric indicators,^9^ and electrochemistry.^10^

Increases in sensitivity are desirable for dilute and trace-level samples. Field-driven concentration methods include isotachophoresis, isoelectric focusing, field amplified sample stacking, and ion concentration polarization.^11,12^ Solvent evaporation^13,14^ does not require electrodes and has been used in microfluidics for concentration^15,16^ and crystallization.^17,18^ The related problem of permeation has been investigated,^19,20^ and pervaporation has been used to explore phase diagrams.^21–25^ Evaporation-driven concentration has also been demonstrated on paper.^26–28^ Evaporation may be enhanced using airflow, and capillary flow may be halted in extreme cases, allowing separation when flow resumes.^29^ Similar evaporation-driven effects also occur in fluidic self-assembly^30^ and in chloride ion concentration in concrete.^31^

Paper diagnostic devices overwhelmingly use linear flow, but the potential advantages of other geometries appear to have escaped detailed attention, despite the observation of enhanced concentration at tips in star- and wedge-shaped layouts.^26,27^ Ignoring boundary effects, a wedge-shaped substrate is a sector of a radial system. Radial flow has long been used in chromatography^32^ and leads to the well-known “coffee stain” effect when combined with evaporation.^33,34^ Radial flow has, therefore, been of considerable interest,^35,36^ and chromatography theory^37^ has been adapted to radial geometries.^38^ Similar radial dispersion problems have also been studied in hydrology.^39,40^

This paper compares paper-based concentration using linear and radial flow. In each case, the main difficulties are that analytic solutions to advection–dispersion equations (ADEs) are difficult to obtain, and repeatable experiments are hard to perform. Both problems are tackled; new analytic solutions are found, and experiments are developed to demonstrate the major effects occurring. The arrangement of the paper is as follows. In Sec. II, approximate, linear ADEs for are presented in 1D and 2D. In Sec. III, numerical results for infinite sources are presented, a new method for finding approximate analytic solutions is proposed, and the theoretical advantages of radial concentration are established. Some limiting assumptions are then revisited. In Sec. IV, details of materials and equipment for fully radial concentration are given. Concentration of dye from a perimeter source is then demonstrated on filter paper under ambient conditions in a reverse of the coffee stain experiment, using optical transmission to quantify concentration. In Sec. V, details of equipment for airflow-enhanced concentration are given. Concentration in strips and wedges on laser-patterned chromatography paper are then compared. In each case, experimental results are matched with theory. Conclusions are drawn in Sec. VI.

## II. ADVECTION–DISPERSION EQUATIONS

In this section, we present the equations for evaporation concentration in linear and radial geometries. The analysis follows microfluidic models,^24^ with adaption to a porous substrate^29^ following the standard chromatography theory.^37^ The models are highly simplified, with the aim of generating analytic solutions that highlight the effect of geometry in the linear regime.

### A. Geometries

**FIG. 1.**
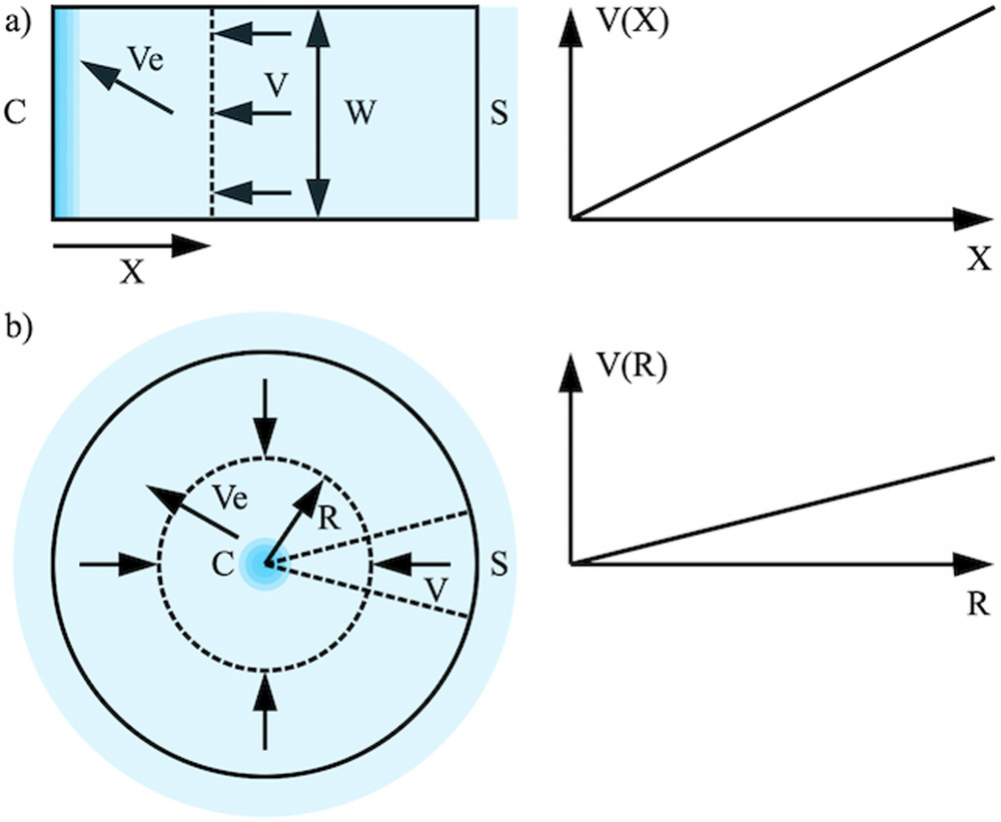
Geometry (LH) and velocity profiles (RH) for (a) linear and (b) radial concentration.

The left-hand sides of Figs. 1(a) and 1(b) show linear and radial concentration. In each case, a non-volatile solute is carried by a volatile solvent from a source *S* to a concentration point *C*, where the flow stagnates, with solvent evaporating between. We assume that the substrates are horizontal, so gravitational effects may be neglected, and ignore the third dimension. A strip-shaped substrate should behave as half of a long strip fed from sources at either end. Similarly, a wedge-shaped substrate should behave like a circular substrate. Boundary effects might be expected; however, we ignore these for simplicity. For practical reasons, a feed section may be needed to connect the source. It is simple to incorporate one;^25^ however, we omit this refinement on the grounds that it will be short.

### B. 1D concentration

Usually, concentration is preceded by wetting, and many authors have considered capillary flow in porous media.^41–44^ However, with moving boundary conditions, it is difficult to solve ADEs and to measure concentration optically. We, therefore, assume that the substrate is fully wetted at the start. This assumption is not unrealistic; wetting takes minutes, while concentration can take hours. In this regime, flow is no longer driven by capillary effects, but by evaporation and the tension of water.

We also assume a uniform evaporation rate, allowing the flow of solvent and solute to be decoupled. This simplification is supported by more detailed models of evaporation from liquid streams and porous media^45–47^ and is common in models of permeation^19,20^ and pervaporation.^21–25^ An exception is work by Schindler and Adjari,^24^ who developed balance equations for binary mixtures. However, they noted that the effect of coupling is to render the evaporation rate, velocity, and diffusion coefficient all functions of solute concentration. The analysis presented here is, therefore, valid only for dilute solutions.

If the substrate has porosity ε and thickness *d*, and the total evaporation flux from both sides is $v_{e}$ per unit area, solvent continuity implies that the velocity *V* in the −X direction is

$$V=\left(\frac{v_{e}}{d}\right)X=\left(\frac{1}{\tau_{e}}\right)X.$$ (1)

Here, *X* is the distance from *C* and $\tau_{e}=d/v_{e}$ is a characteristic time constant. This variation is linear, as shown in the right-hand side of Fig. 1(a), falling to zero at *C*.

We now consider the transport of a single solute with concentrations $C_{L}$ and $C_{S}$ in the liquid and solid phases. Although diffusive dynamics are known to cause a non-Fickian form of diffusion in channels,^48^ porous media,^49,50^ and micro-structured media,^51,52^ described by velocity-dependent axial and transverse diffusion coefficients, we assume a constant scalar *D* for simplicity. We also ignore the change in *D* resulting from the cooling effect of evaporation, which may be estimated via the Stokes–Einstein relation. For a flow velocity *U* in the +X direction, the continuity equation is

$$\frac{\partial C_{L}}{\partial T}+F\frac{\partial C_{S}}{\partial T}=D\frac{\partial^{2}C_{L}}{\partial X^{2}}-\frac{\partial (UC_{L})}{\partial X}.$$ (2)

Here, *T* is the time, and F=(1−ε)/ε is the volumetric ratio of the stationary and mobile phases. The LHS describes accumulation, and the RHS models describe diffusion and transport. Substituting U=−V, we obtain

$$\frac{\partial C_{L}}{\partial T}+F\frac{\partial C_{S}}{\partial T}=D\frac{\partial^{2}C_{L}}{\partial X^{2}}+\left(\frac{X}{\tau_{e}}\right)\frac{\partial C_{L}}{\partial X}+\left(\frac{1}{\tau_{e}}\right)C_{L},$$ (3)

where $C_{S}$ and $C_{L}$ are related by the adsorption isotherm $C_{S}\;=\;f(C_{L})$, which depends on the adsorption mechanism. For a single solute, common models include linear, Langmuir (saturating), Freundlich, and BET isotherms.^53^ For the linear isotherm, $C_{S}=aC_{L}$, where *a* is Henry's coefficient. In this case, the continuity equation becomes

$$\frac{1}{R_{f}}\frac{\partial C_{L}}{\partial T}=D\frac{\partial^{2}C_{L}}{\partial X^{2}}+\left(\frac{X}{\tau_{e}}\right)\frac{\partial C_{L}}{\partial X}+\left(\frac{1}{\tau_{e}}\right)C_{L}.$$ (4)

Here, $R_{f}=1/\left(1+aF\right)$ is the retardation factor. We now introduce a normalized time $t=T/\tau_{e}$ and a normalized position $x=X/X_{M}$, where $X_{M}$ is the substrate length to get

$$\frac{\partial C_{L}}{\partial t^{\prime }}=\frac{1}{Pe}\frac{\partial^{2}C_{L}}{\partial x^{2}}+x\frac{\partial C_{L}}{\partial x}+C_{L}.$$ (5)

Here, $t^{\prime }=R_{f}t$ and $Pe=\frac{X_{M}^{2}}{D\tau_{e}}$ is the Péclet number.^54^ A large value of Pe ensures that advection can overcome back-diffusion to achieve a locally high solute concentration. This ADE is more complex than the well-studied form with uniform velocity and constant coefficients. However, it corresponds to previous models for microfluidic concentration and is extensible to nonlinear and competitive adsorption.

### C. 2D concentration

We now consider the ADE in the radial geometry. For inward radial flow and uniform evaporation, the solvent velocity profile is

$$V=\left(\frac{1}{\tau_{e}}\right)\frac{R}{2}.$$ (6)

Here, *R* is the distance from *C*. This variation is again linear, as shown in the right-hand side of Fig. 1(b); however, the maximum velocity is half that obtained for a strip substrate. For a variable flow velocity *U* in the +R direction, the solute continuity equation is

$$\begin{array}{rl} \frac{\partial C_{L}}{\partial T}\;+\;F\frac{\partial C_{S}}{\partial T} & \;=\;D\left\{\;\frac{\partial^{2}C_{L}}{\partial R^{2}}+\left(\frac{1}{R}\right)\frac{\partial C_{L}}{\partial R}\right\}\\ & \;\;-\left\{\frac{\partial (UC_{L})}{\partial R}+\left(\frac{1}{R}\right)UC_{L}\right\}. \end{array}$$ (7)

Substituting U=−V, introducing a normalized time and a normalized position $r=R/R_{M}$, where $R_{M}$ is the substrate radius, and assuming a linear isotherm, we obtain

$$\frac{\partial C_{L}}{\partial t^{\prime }}=\;\frac{1}{Pe}\left(\frac{1}{r}\right)\frac{\partial }{\partial r}\left(r\frac{\partial C_{L}}{\partial r}\right)+\left(\frac{r}{2}\right)\frac{\partial C_{L}}{\partial r}+C_{L}.$$ (8)

Here, $Pe=\frac{R_{M}^{2}}{D\tau_{e}}$ is the radial Péclet number.

The linear and radial ADEs are different and, hence, must yield different concentration profiles. In each case, $C_{S}$ can be found from the isotherm once $C_{L}$ is known, and the average concentration $C_{AV}$ (which is optically measurable) is evaluated as $C_{AV}=\varepsilon C_{L}+(1-\varepsilon )C_{S}$. For linear adsorption, $C_{AV}=kC_{L}$, where k=ε+a(1−ε).

ADEs with constant coefficients have been solved by change of variables, Laplace transforms and numerical integration, and solutions are known in hydrology for many boundary conditions and initial conditions.^55^ The main difficulties are to identify substitutions, invert transforms, and integrate stiff equations. Less attention has been paid to ADEs with spatially variable coefficients, although solutions exist for fortuitous combinations arising from Taylor dispersion.^56,57^ Analytic approximations^21–25^ and numerical solutions^24^ have also been presented for the linear and nonlinear cases, respectively.

## III. SOLUTION OF THE ADEs

In this section, we first provide numerical solutions to the ADEs and review existing approximations. We then develop new approximations for the full concentration profile.

### A. Numerical solutions and approximations

**FIG. 2.**
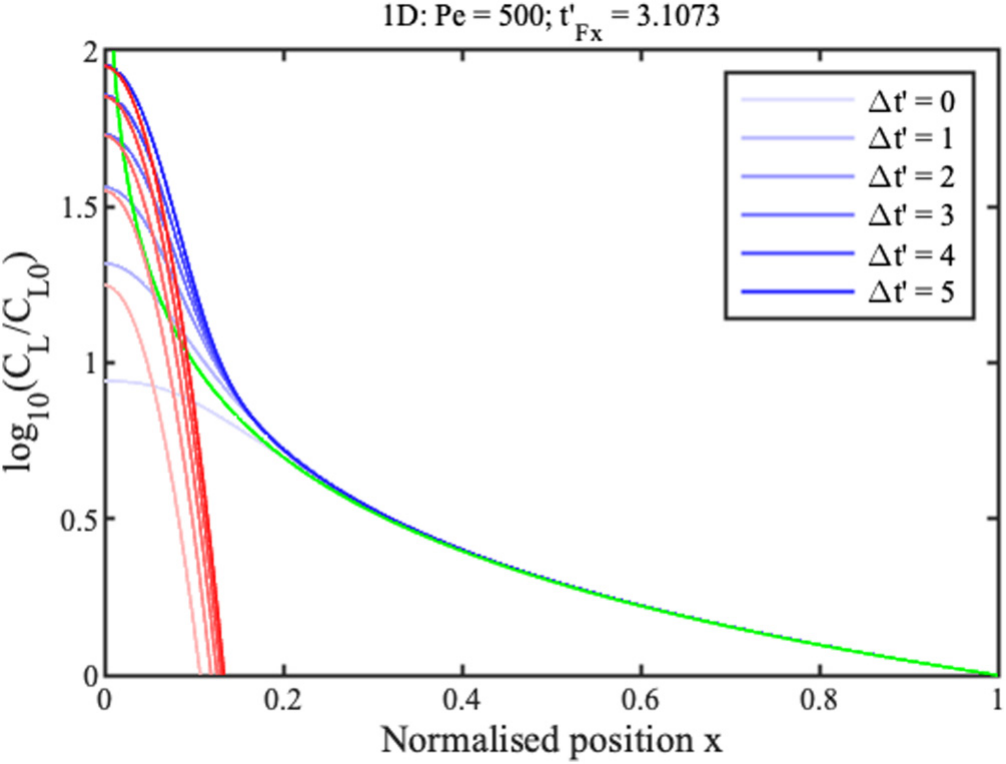
Theoretical profiles for 1D concentration. Blue lines show numerical results. Green and red lines show approximations for the ramp and concentration peak.

**FIG. 3.**
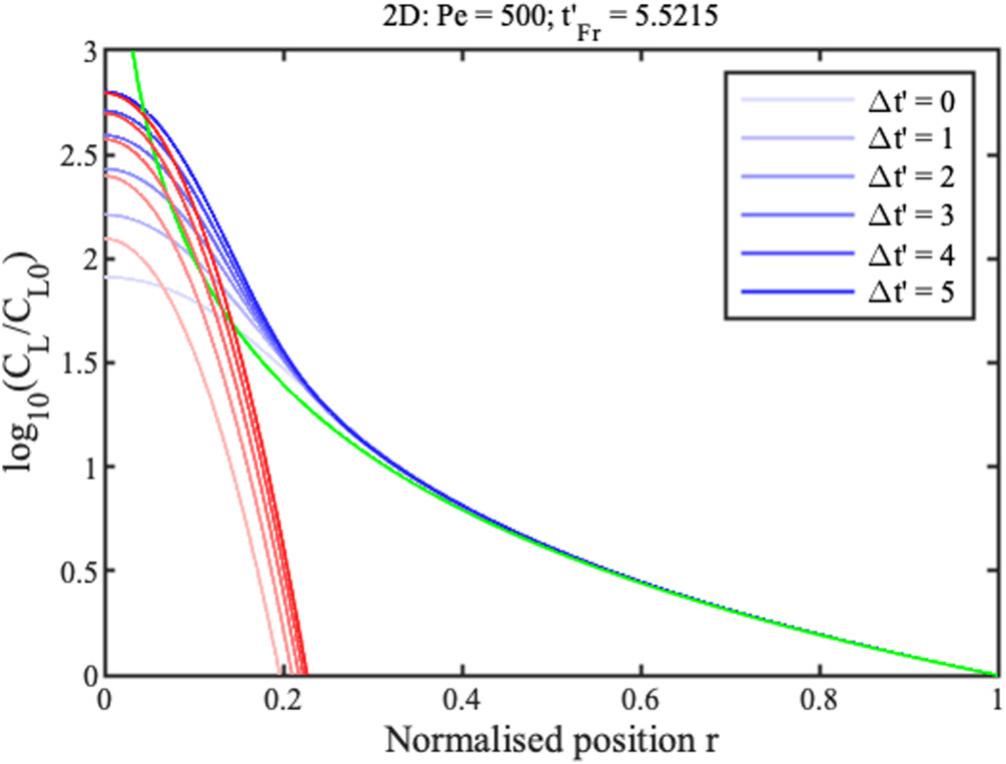
Theoretical profiles for 2D concentration. Blue lines show numerical results. Green and red lines show approximations for the ramp and concentration peak.

Assuming abrupt connection to a reservoir at concentration $C_{L0}$, the boundary conditions in the linear case are $C_{L}(x,0)=0$; $C_{L}(1,t^{\prime })=C_{L0}$ and $\partial C_{L}/\partial x{|}_{x=0}=0$ and the solution requires integration over 0≤x≤1, $0\leq t^{\prime }t_{max}^{\prime }$. Boundary conditions are similar in the radial case, replacing *x* with *r*. Numerical solutions are obtained in Matlab using the function “pdepe,” which can integrate parabolic and elliptic partial differential equations. The blue lines in Figs. 2 and 3 show concentration profiles on a logarithmic scale at different normalized times $\Delta t^{\prime }=t^{\prime }-t_{F}^{\prime }$ for 1D and 2D concentration, respectively, assuming that Pe=500. Here, $t_{F}^{\prime }$ is the filling time, discussed below.

Near the reservoir (x,r≫0) all curves tend to a steady profile. In this region, approximate solutions $C_{Lx}$ and $C_{Lr}$ for the two cases can be obtained by neglecting time variation and diffusion, as

$$\frac{C_{Lx}}{C_{L0}}\approx \frac{1}{x};\;\;\frac{C_{Lr}}{C_{L0}}\approx \frac{1}{r^{2}}.$$ (9)

These solutions are shown in green in Figs. 2 and 3. They match the numerical results well in the ramp regions but poorly elsewhere. The solution for the 1D geometry is known as the hyperbolic ramp.^21–24^ In each case, concentration rises gradually with distance from the reservoir, but unfortunately tends to infinity at the origin. The rise is clearly faster for the radial case.

Near the origin (x,r≪1), the profiles are Gaussian, with a peak value that increases linearly with time. Here, the following approximate solutions are valid,

$$\begin{array}{r} \frac{C_{Lx}}{C_{L0}}\approx (t^{\prime }-{t^{\prime }}_{Fx})\surd \left(\frac{2Pe}{\pi }\right)e^{-x^{2}Pe/2},\\ \frac{C_{Lr}}{C_{L0}}\approx (t^{\prime }-{t^{\prime }}_{Fr})\frac{Pe}{4}e^{-r^{2}Pe/4}.\;\;\; \end{array}$$ (10)

Here, $t_{Fx}^{\prime }$ and $t_{Fr}^{\prime }$ are normalized filling times. Once again, the solution for the 1D geometry is already known.^21^ These solutions are shown in red in Figs. 2 and 3. They match the numerical profiles near the concentration peak but are clearly inaccurate elsewhere. The peak is clearly wider in the radial case but, despite this, concentration factors are much larger.

**FIG. 4.**
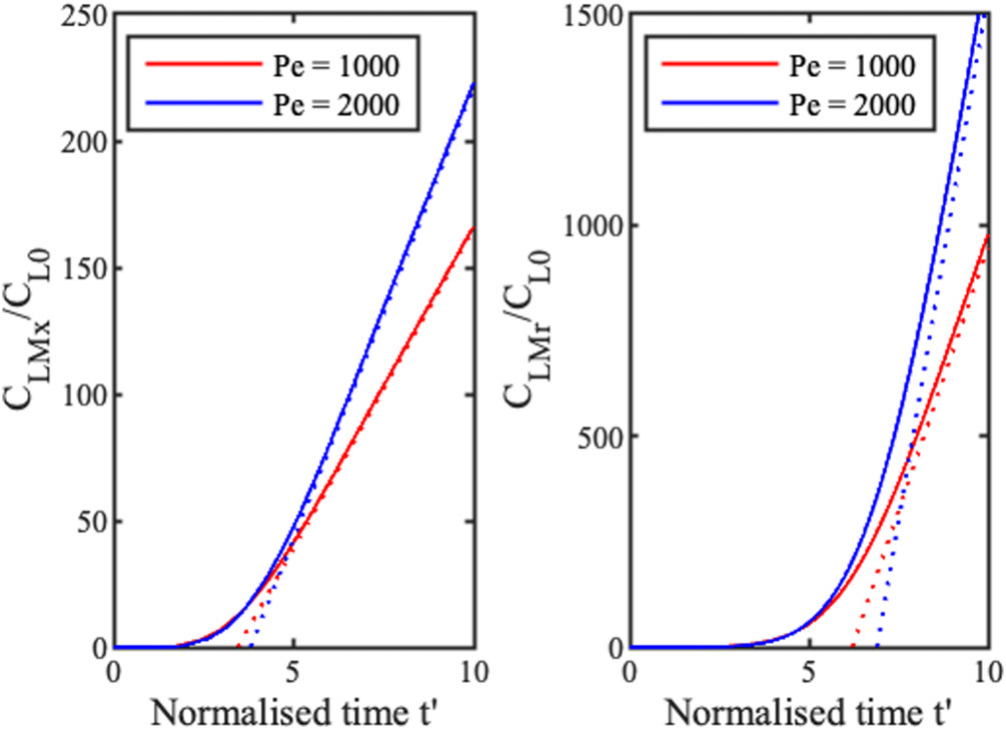
Variations of peak concentration with normalized time for 1D (LH) and 2D (RH) concentration. Full lines show numerical results, and dotted lines show analytic approximations.

Filling times may be estimated by matching the peak concentrations $C_{LMx}=\max(C_{Lx})$ and $C_{LMr}=\max(C_{Lr})$ from Eq. (10) to numerical results. The full and dotted lines in Fig. 4 show numerical and analytic variations for 1D (LH) and 2D (RH) concentration and two values of Pe. In each case, the numerical variations gradually tend to the linear approximations, and the longer filling times but larger concentration rates achieved in the radial case should be noted.

**FIG. 5.**
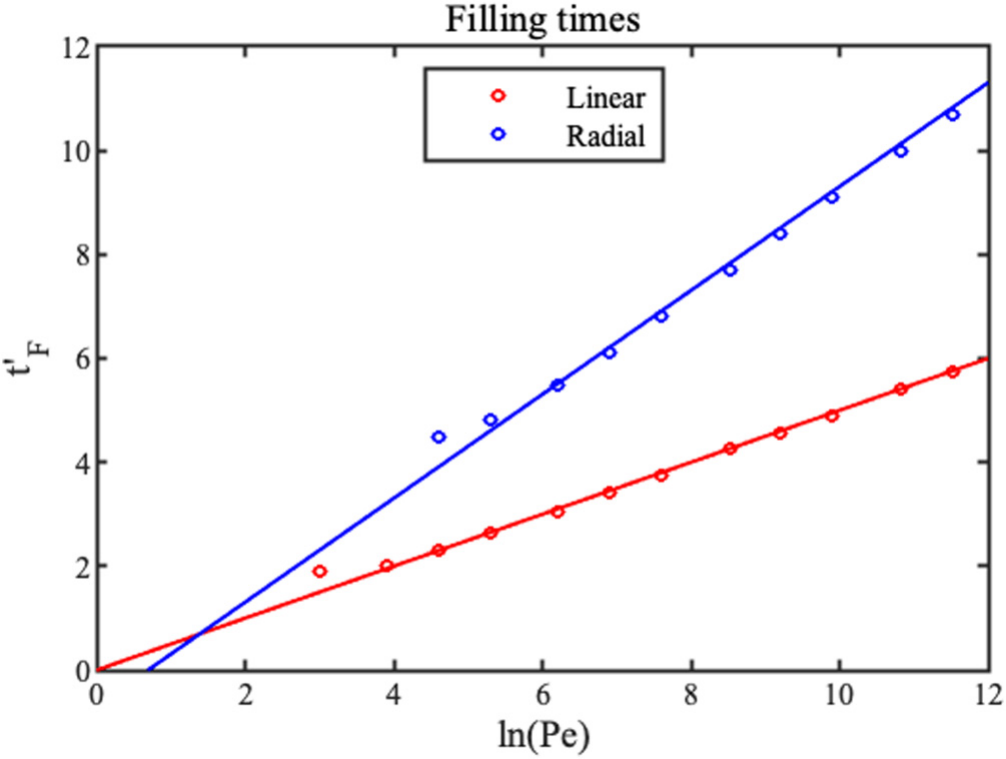
Variation of 1D and 2D filling times with ln(Pe). Points show estimates from numerical solutions; lines show analytic estimates.

The points in Fig. 5 show the variations with ln⁡(Pe) of normalized filling time obtained by this matching, together with straight-line fits that allow the following analytic estimates:

$$t_{Fx}^{\prime }\approx \ln\left(\sqrt{Pe}\right);\;\;t_{Fr}^{\prime }\approx \ln(Pe/2).$$ (11)

Again, the expression for $t_{Fx}^{\prime }$ is known.^25^ The estimates are accurate unless Pe is small. For Pe>4 (a trivial value), the filling time is longer for the radial case.

### B. New analytic approximations

The approximations above are not satisfactory since they do not allow complete concentration profiles being drawn. We now develop new solutions that avoid this problem. To do so, we assume at the outset that the profile after filling can be written as the sum of time-varying and static parts. In the linear case, we, therefore, write for $t^{\prime }>t_{Fx}^{\prime }$,

$$C_{Lx}(x,t^{\prime })\approx (t^{\prime }-{t^{\prime }}_{Fx})C_{1}(x)+C_{2}(x).$$ (12)

Substitution into the ADE then yields the ordinary differential equation,

$$\begin{array}{r} C_{1}\approx (t^{\prime }-{t^{\prime }}_{Fx})\left\{\frac{1}{Pe}\frac{d^{2}C_{1}}{dx^{2}}+x\frac{dC_{1}}{dx}+C_{1}\right\}+\frac{1}{Pe}\frac{d^{2}C_{2}}{dx^{2}}+x\frac{dC_{2}}{dx}+C_{2}. \end{array}$$ (13)

Since the time-dependent terms must vanish separately, $C_{1}$ must satisfy

$$\frac{1}{Pe}\frac{d^{2}C_{1}}{dx^{2}}+x\frac{dC_{1}}{dx}+C_{1}=0.$$ (14)

Direct substitution shows that

$$\frac{C_{1}(x)}{C_{L0}}=A\;\exp\left(-Pe\frac{x^{2}}{2}\right).$$ (15)

To match the filling rate for unit strip width, solute conservation requires that $\int_{0}^{1}C_{1}\;dx=C_{L0}\cdot 1$ so that $A=\sqrt{2Pe/\pi }$. This result implies that the time-varying solution corresponds exactly to the upper of Eq. (10). The function $C_{2}$ must then satisfy

$$\frac{1}{Pe}\frac{d^{2}C_{2}}{dx^{2}}+x\frac{dC_{2}}{dx}+C_{2}=C_{L0}\sqrt{\frac{2Pe}{\pi }}\;\exp\left(\frac{-x^{2}Pe}{2}\right).$$ (16)

Surprisingly, this inhomogeneous equation can be integrated directly. Assuming the boundary conditions $C_{2}=\frac{\partial C_{2}}{\partial x}=0$ on x=0, we obtain

$$\frac{C_{2}(x)}{C_{L0}}=\;Pe\;\exp\left(\frac{-x^{2}Pe}{2}\right)\int_{0}^{x}\mathrm{erf}\left(y\sqrt{\frac{Pe}{2}}\right)\;\exp\left(\frac{y^{2}Pe}{2}\right)dy.$$ (17)

It is simple to show that this result approximates a hyperbola as x→1, but the overall variation is now a realistic bounded function that tends to zero as x→0.

Performing a similar procedure for the radial case but now assuming that $C_{Lr}(r)\approx (t^{\prime }-{t^{\prime }}_{Fr})C_{1}(r)+C_{2}(r)$ for $t^{\prime }\geq t_{Fr}^{\prime }$ and requiring that $\int_{0}^{1}C_{1}\;2\pi r\;dr=\;C_{L0}\cdot 2\pi r\cdot 1/2\;$ (where the factor of ½ arises from the radial velocity profile) leads to

$$\frac{C_{1}(r)}{C_{L0}}=\frac{Pe}{4}\exp\left(\frac{-rPe^{2}}{4}\right).$$ (18)

This result implies that the time-varying solution corresponds exactly to the lower of Eq. (10). Substitution and integration then yields

$$\frac{C_{2}(r)}{C_{L0}}=\left(\frac{Pe}{4}\right)\exp\left(\frac{-r^{2}Pe}{4}\right)\left\{\;\mathrm{ei}\left(\frac{r^{2}Pe}{4}\right)-\ln\left(\frac{r^{2}Pe}{4}\right)-\gamma \right\}.$$ (19)

Here, $\mathrm{ei}(x)=\int_{-\infty }^{x}\frac{e^{t}}{t}dt$ is the exponential integral and γ≈0.57721 is the Euler–Mascheroni constant. Equation (19) tends to $1/r^{2}$ as r→1, but the overall variation has again been replaced by a bounded function.

**FIG. 6.**
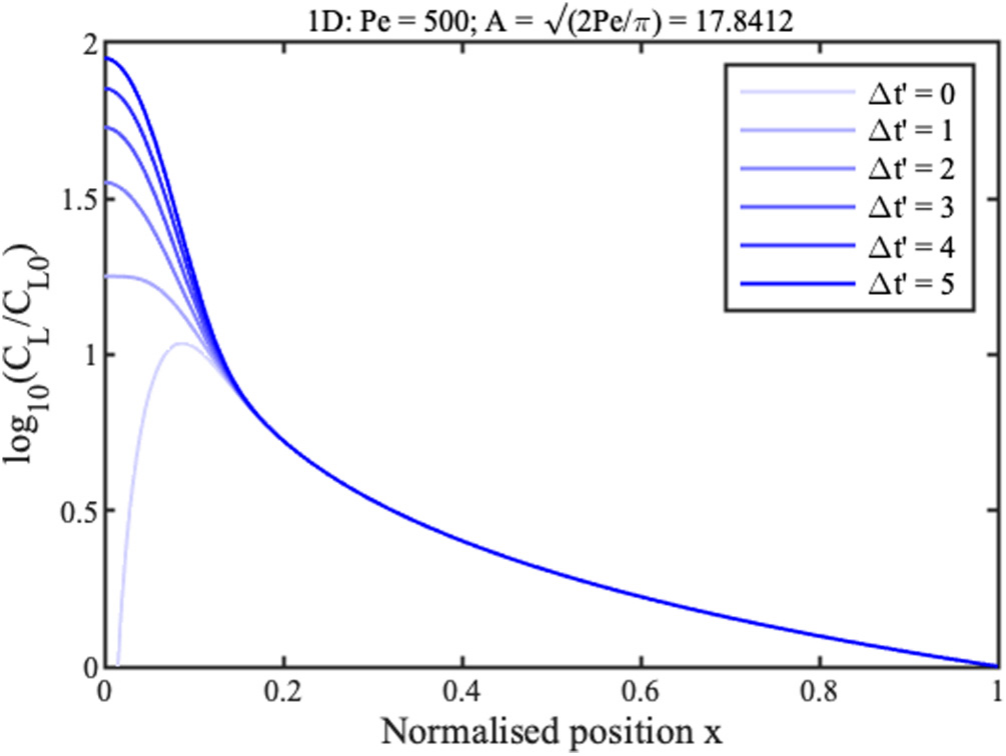
New approximate theoretical profiles for 1D concentration, at different normalized times.

**FIG. 7.**
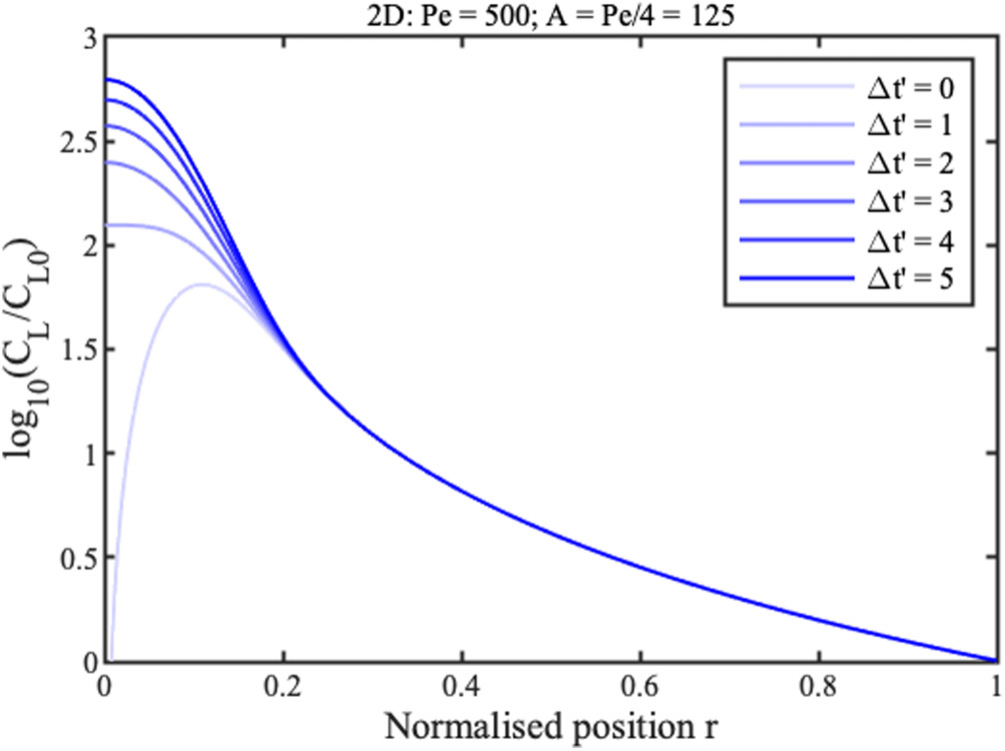
New approximate theoretical profiles for 2D concentration, at different normalized times.

Figures 6 and 7 show these analytic solutions at different normalized times $\Delta t^{\prime }$ for linear and radial concentration, respectively, again assuming that Pe=500. These results should be compared with Figs. 2 and 3.

The new solutions present physically realistic approximations for the entire concentration profile and (despite some inaccuracy for small $\Delta t^{\prime }$) tend to the numerical variations as $\Delta t^{\prime }$ increases.

Using the analytic solutions, the filling times may be estimated as

$$t_{Fx}^{\prime }=\frac{\int_{0}^{1}C_{2}\;dx}{\int_{0}^{1}C_{1}d\;x};\;\;t_{Fr}^{\prime }=\frac{\int_{0}^{1}C_{2}\;2\pi r\;dr}{\int_{0}^{1}C_{1}\;2\pi r\;dr}.$$ (20)

Numerical integration shows that these expressions agree well with Eq. (11) and Fig. 5.

We have not been able to perform an analytic integration; however, we note that Eqs. (17) and (19) may be approximated as

$$\frac{C_{2}(x)}{C_{L0}}\approx \frac{1-e^{-{\left(x\sqrt{Pe/2}\right)}^{3}}}{x};\;\;\frac{C_{2}(r)}{C_{L0}}\approx \frac{1-e^{-\frac{1}{\sqrt{2}}{\left(Pe\frac{r^{2}}{4}\right)}^{2}}}{r^{2}}.$$ (21)

These expressions highlight the limiting behavior near the source and stagnation point more clearly. Usefully, they may both be integrated analytically, to yield

$$\begin{array}{rl} & \;t_{Fx}^{\prime }\approx \ln\left(\sqrt{Pe}\right)+\left\{\frac{\gamma }{3}-\frac{\ln(2)}{2}\right\};\\ & t_{Fr}^{\prime }\approx \ln\left(\frac{Pe}{2}\right)+\left\{\frac{\gamma }{2}-\frac{\ln\left(4\sqrt{2}\right)}{2}\right\}. \end{array}$$ (22)

In each case, the first term may be recognized as the corresponding filling time in Eq. (11), confirming the dependence on Pe. The second term is a small constant error, which has the numerical value −0.1541 in the 1D case and −0.5778 in the 2D case.

Although the 1D geometry has a shorter filling time, a flatter ramp, and a sharper concentration peak, these results imply that the radial geometry concentrates more quickly after filling. In fact, the ratio of the peak values $C_{LMr}$ and $C_{LMx}$ for $t^{\prime }\gg t_{F}^{\prime }$ is

$$\frac{C_{LMr}}{C_{LMx}}\approx \sqrt{\frac{\pi Pe}{32}}.$$ (23)

This ratio is unity for Pe≈10 and 10 for Pe≈1000. Radial-flow evaporation concentrators are, therefore, fundamentally more effective than linear concentrators, and this advantage is clear in the earlier Fig. 4. The only uncertainty is whether a suitably high Pe can be achieved experimentally before permeability effects limit capillary rise to an intermediate stagnation point.^29^ We now confirm this is the case.

### C. Model limitations

**FIG. 8.**
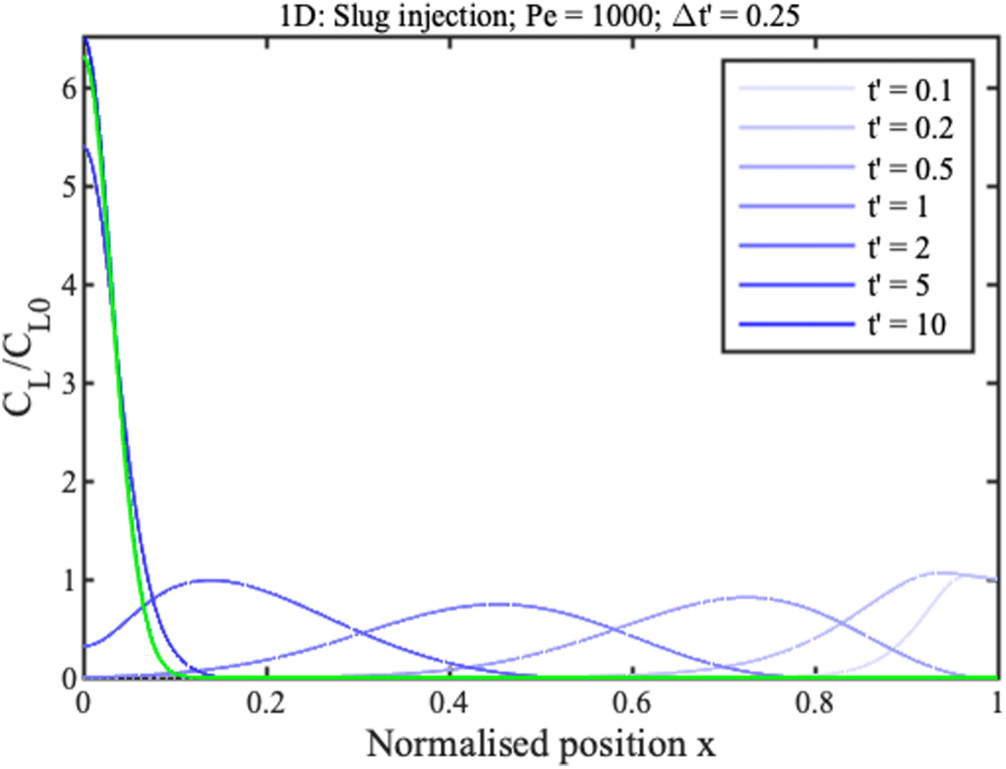
Theoretical profiles for 1D concentration at different normalized times assuming injection of a finite slug followed by pure solvent (blue); steady-state peak shape (green).

We now consider three limitations of the model. First, the analyte supply is likely to be finite. ADEs can always be solved numerically for such cases. For example, the blue lines in Fig. 8 show concentration profiles at different times for a 1D concentrator with Pe=1000, assuming injection of an analyte slug for a time $\Delta t^{\prime }=0.25$ followed by pure solvent. Solute may be seen entering from the source until $t^{\prime }=\Delta t^{\prime }$; after this, it travels to the origin by solvent pumping. The concentration peak now tends to a steady state, which from previous analysis must clearly be $C_{Lx}\approx \Delta t^{\prime }C_{1}(x)$. The prediction of this expression is shown in green in Fig. 8; the agreement with the final numerical curve is exact. A similar result is obtained for radial flow, confirming retention of its geometric advantage for a finite supply.

Second, the velocity spread inherent in porous media results in Taylor dispersion, often modeled by an effective axial diffusion coefficient $D_{a}=D(1+\kappa V^{m})$, where *D* is the molecular coefficient, *V* is the velocity, and κ and *m* are constants. For the velocity profile in a 1D concentrator, this can be written as $D_{a}=D(1+\alpha x^{m})$, where $\alpha =\kappa {\left(X_{M}/\tau_{e}\right)}^{m}$. The effect of a varying diffusion coefficient $D_{a}=Df(x)$ is then to modify the ADE to

$$\frac{\partial C_{L}}{\partial t^{\prime }}=\left(\frac{1}{Pe}\right)\frac{\partial }{\partial x}\left(\;f\frac{\partial C_{L}}{\partial x}\right)+x\frac{\partial C_{L}}{\partial x}+C_{L}.$$ (24)

The previous method allows solutions for $C_{1}$ and $C_{2}$ to be found for the infinite source case, for both m=1 and m=2. The expressions are complicated, so here we give only those for $C_{1}$,

$$m=1;\;\;C_{1}=A\;e^{Pe\frac{\left\{\log(1+\alpha x)-\alpha x\right\}}{\alpha^{2}}},$$ (25)

$$m=2;\;\;C_{1}=\frac{A}{{\left(\alpha x^{2}+1\right)}^{\frac{Pe}{2\alpha }}}.$$ (26)

**FIG. 9.**
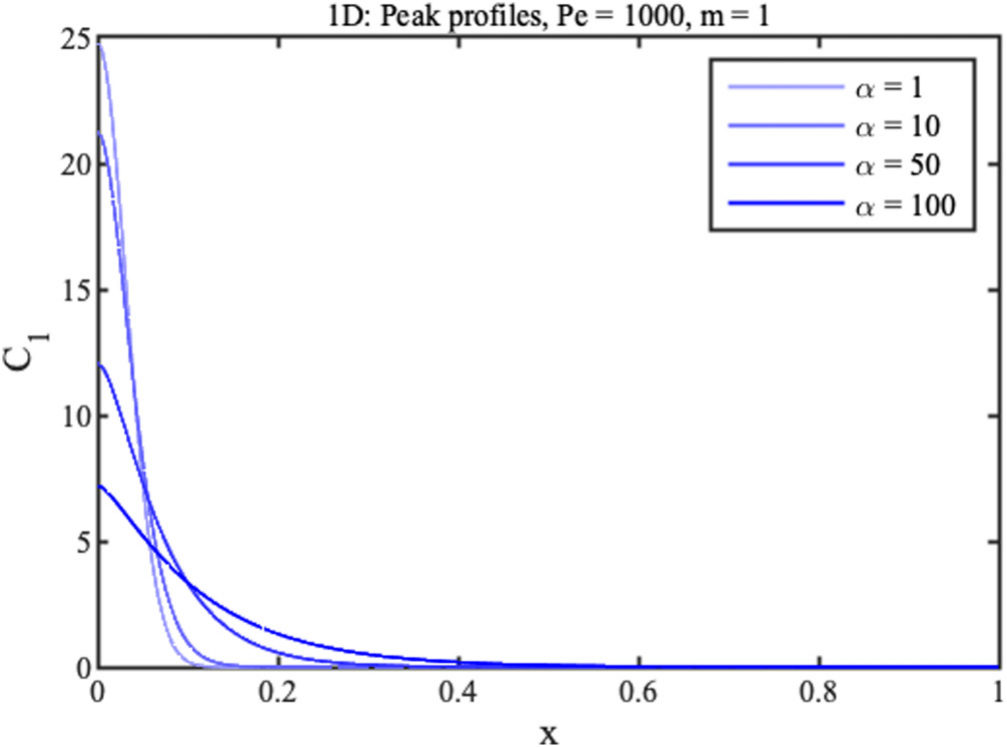
Peak profiles for 1D concentration with Pe=1000, m=1, and different values of the Taylor coefficient α.

Here, *A* is a constant chosen to conserve the quantity of solute in the peak and is most easily found by numerical integration. Figure 9 shows this solution for Pe=1000,m=1, and different α.

The effect of increasing α is to broaden the concentration peak and reduce the concentration rate. However, because the velocity is inherently low near the origin, large values are required to make a significant difference. A similar ADE may be constructed for radial concentrators; this can be solved numerically, but we have so far failed to find analytic solutions.

Third, non-uniform evaporation may give rise to a nonlinear normalized flow velocity profile g(x). In this case, the ADE modifies to

$$\frac{\partial C_{L}}{\partial t^{\prime }}=\frac{1}{Pe}\frac{\partial^{2}C_{L}}{\partial x^{2}}+\frac{\partial (gC_{L})}{\partial x}.$$ (27)

The previous method again allows solutions for $C_{1}$ and $C_{2}$ to be found for the infinite source case. Assuming the polynomial variation $g(x)=a_{1}x+a_{2}x^{2}+a_{3}x^{3}\cdots$ the solution for $C_{1}$ is the modified Gaussian concentration profile,

$$C_{1}=A\exp\left\{-Pex^{2}\left(\frac{a_{1}}{2}+\frac{a_{2}}{3}x+\frac{a_{3}}{4}x^{2}\ldots \right)\right\}.$$ (28)

For evaporation profiles varying mainly near the source, the effect on the peak shape is also small.

## IV. RADIAL CONCENTRATION

In this section, we demonstrate radial concentration using water-soluble dye on filter paper disks. There are several experimental difficulties. The first is to provide a peripheral source, and a repeatable geometry was achieved by paper cutting on a jig. The second is to measure dye concentration. This can be estimated from optical transmission. However, variations due to changes in paper morphology are difficult to avoid. Measurements were, therefore, only carried out when substrates were mechanically stabilized. The third is to obtain a suitable dynamic range of concentration. Trials were, therefore, carried out with different dye concentrations. The last is to control ambient conditions. Temperature and humidity are unrepeatable without an environmental chamber. These parameters were, therefore, simply monitored as in the range 20–22 °C and 45–65% RH using a temperature and humidity meter (Type 971, Fluke).

### A. Materials

Concentration was carried out on 90 mm diameter Qualitative Circles (1001090, Whatman, Little Chalfont, UK, thickness 180μm, pore size 11μm). The solute was the water-soluble triphenylmethane dye Brilliant Blue FCF (erioglaucine disodium salt or C_37_H_34_N_2_Na_2_O_9_S_3_, also known as acid blue 9, E133, and CI 42090), used as a food dye and as a tracer in soil science. Its diffusion coefficient is known to be $D=5.68\times 10^{-10}\;m^{2}/s$.^58^ Dye was obtained at ≥97% purity (80717, Sigma Aldrich, St. Louis, USA) and dissolved in de-ionized water to form stock solutions. Its retardation factor on Whatman paper is quoted as close to unity,^34^ and we have verified it as $R_{f}\approx 0.98$.^29^

**FIG. 10.**
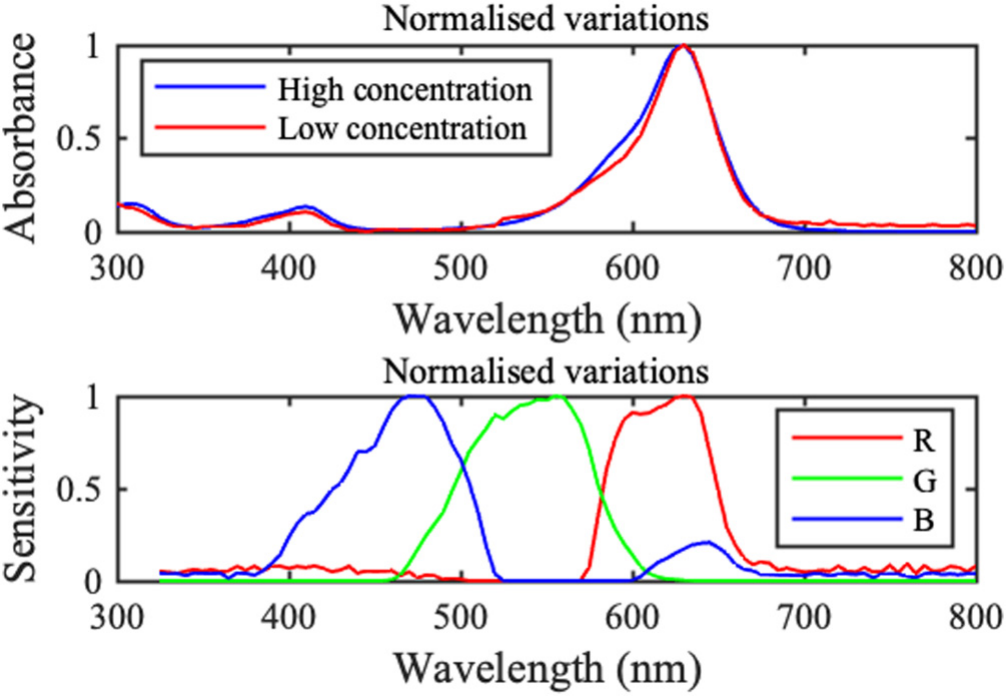
Spectral variation of absorbance for Brilliant Blue (upper) and sensitivity for Dino-Lite image sensor (lower).

Absorbance was measured over a wide concentration range using an Ultrospec III UV-visible spectrophotometer (Pharmacia LKB Biotechnology AB, Uppsala, Sweden). The upper plots in Fig. 10 show normalized spectra at low (1.09μg/ml) and high (558μg/ml) concentrations using liquid cells. In each case, absorbance peaks at 630nm. It follows that spatial maps of dye concentration can also be deduced by monitoring the red channel of any imaging sensor producing an RGB image. There are smaller peaks near 310 and 410nm, implying that there will also be smaller changes in the blue channel.

**FIG. 11.**
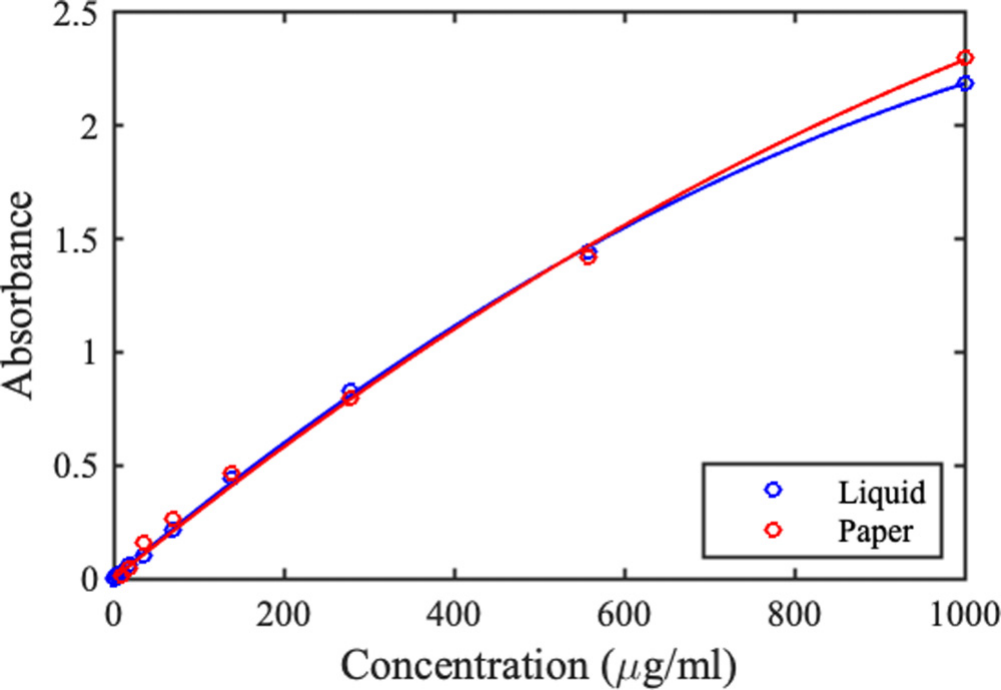
Variation of peak absorbance with concentration for Brilliant Blue.

Figure 11 shows the variation of absorbance at 630 nm with concentration obtained using 0.2 mm thick liquid-filled cells and 0.18 mm thick dye-wetted paper strips, with the former rescaled to match thickness. While some minor nonlinearity is apparent at high concentration, the Beer–Lambert law can be used to deduce concentration from measured absorbance.^8^ The spectra in Fig. 8 are almost identical, and spectra for the liquid-filled cells and wetted paper strips (not shown) are also very similar. Hence, peak shapes and positions are unaffected by dye concentration or a paper substrate.

Concentration experiments were monitored by measuring transmission with a Dino-Lite AM2011 Basic digital microscope (AnMo Electronics Corp., Taipei, Taiwan), using white InGaN-on-sapphire LED striplights for illumination (LuckyLight, Shenzhen, China). The spectral variation in sensitivity of any image sensor is determined by its color filter array (CFA), and the characteristics of the Dino-Lite sensor were investigated by imaging monochromatic light from the spectrophotometer. The lower plots in Fig. 8 show the normalized sensitivities of the red, green, and blue channels, which agree well with the published data for other Bayer pattern CFAs.^59^ The red channel variation is an excellent match to the dye absorption peak; however, all three channels are broadband, and polychromatic measurements are well known to yield apparently nonlinear variations at high absorbance.^60,61^ Very dilute dye solutions (∼0.8μg/ml) were, therefore, used to keep absorbance in the linear regime and light intensity within the dynamic range of the sensor even at high concentration.

### B. Apparatus

**FIG. 12.**
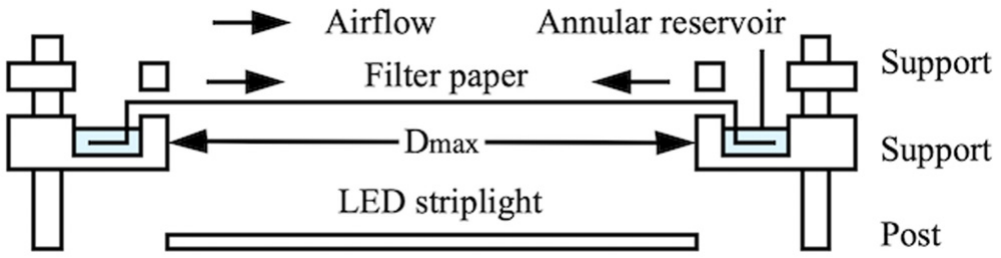
Arrangement for radial concentration.

Figure 12 shows a schematic of the equipment used. The paper was held horizontally between two rapid prototyped supports, designed to allow suspension of a portion with diameter $D_{max}=2R_{max}=60\;\mathrm{mm}$ above a white LED striplight. The lower support contained an annular reservoir linked to the evaporation section by a 5 mm-long non-evaporative feed, and the upper support contained 24 slots and holes to act as scalpel guides and allow dye injection. Gentle airflow from a 22 CFM axial fan (D481T-012KA-3, Micronel AG, Zurich, Switzerland) placed nearby was used to avoid air stagnation. Transmission was measured by time-lapse photography using the digital microscope. Matlab was used to locate the center and radius of the evaporation region in digital images and extract the brightness along a diameter. Averaging and Gaussian smoothing was used to improve signal-to-noise ratio. Relative transmission was obtained by comparison with a reference image and converted to absorbance. Relative dye concentration was then estimated by comparison with calibration measurements.

### C. Results

**FIG. 13.**
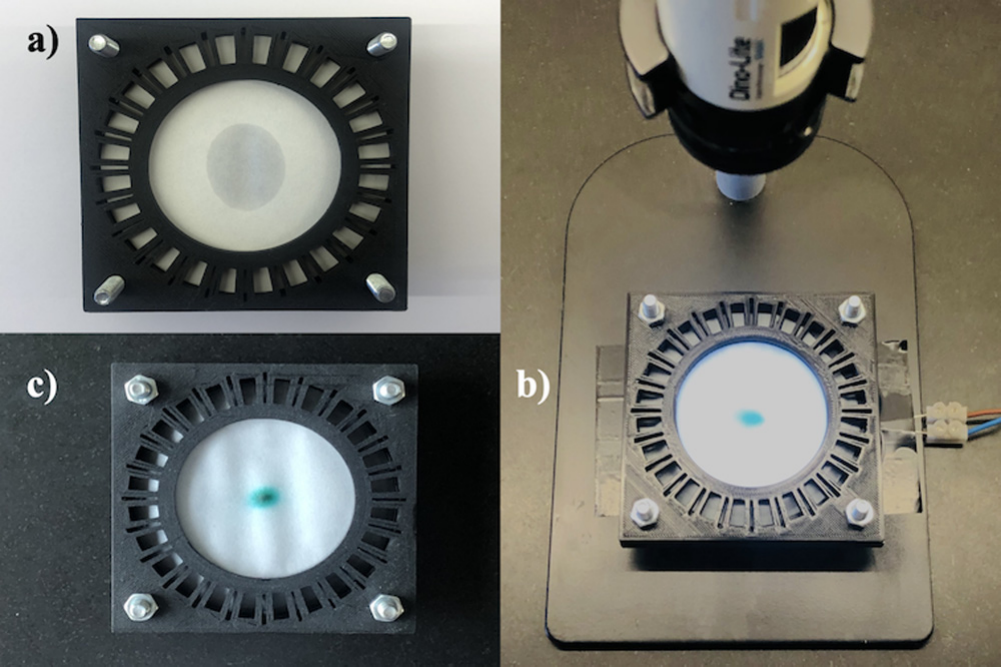
Radial concentrator: (a) identification of the machine direction, (b) complete rig after concentration, and (c) correlation of concentration ellipse with the machine direction.

Exact circular symmetry was impossible to obtain due to anisotropy in the cellulose microstructure, which follows the so-called machine- and cross-directions.^32^ Anisotropic swelling increases expansion in the cross-direction and this, in turn, creates surface wrinkles. Anisotropic permeability then leads to faster capillary filling in the machine direction and elliptical concentration spots, with principal axes parallel and perpendicular to wrinkles. A single drop of water was, therefore, first used to identify the machine direction, and the principal directions were aligned to x- and y-axes of the optical system. Figure 13(a) shows the orientation process; the fast-flow direction is vertical. Scalpel cuts were then made to allow the paper to be folded into the reservoir, and the substrate was wetted. The reservoir was then filled with dye solution and concentration was carried out. Figure 13(b) shows the rig after an experiment; the concentrate is clearly visible at the paper center. Figure 13(c) shows a concentration spot. The ellipse is correlated with paper anisotropy, with the minor axis in the fast-flow direction.

**FIG. 14.**
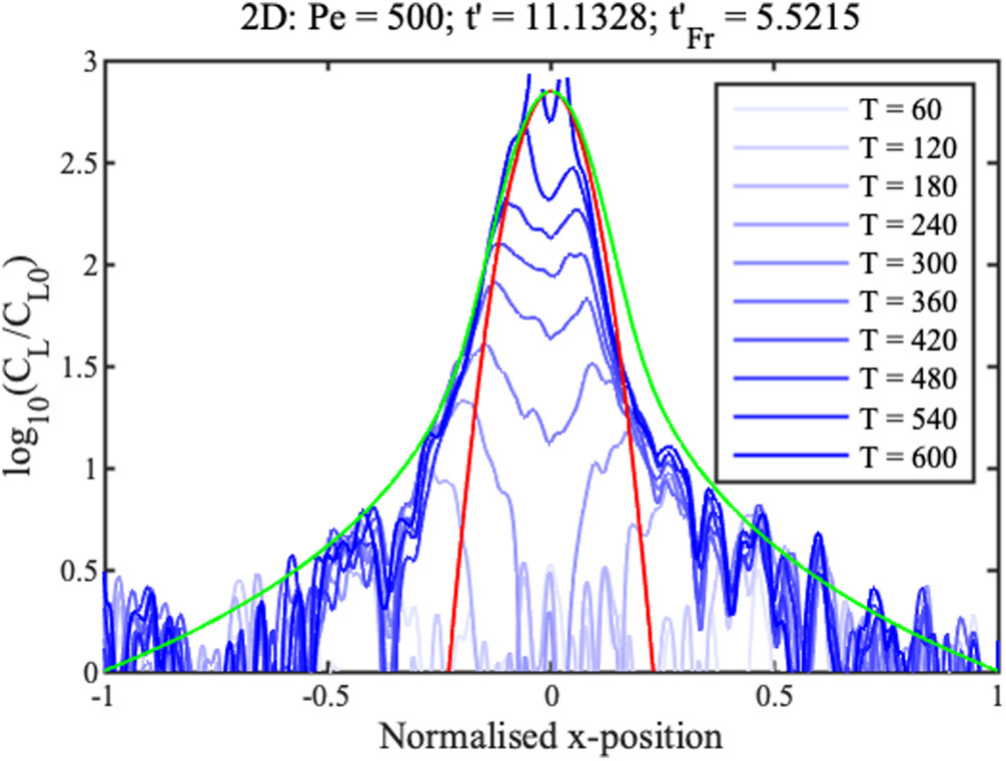
Concentration profiles on filter paper. Blue lines show measured data; red and green lines show theoretical fits to the concentration peak and full profile.

The blue lines in Fig. 14 show typical concentration profiles for the major axis on a log scale at the times shown in minutes up to 10 h. Profiles follow theoretical predictions. There are two peaks during filling, which merge as concentration proceeds. The maximum measurable concentration factor is ∼600, the limit of dynamic range.

Theory was matched to the experiment as follows. There is only one free parameter, the evaporation rate, which determines the time constant $\tau_{e}$. This can be written in terms of the Péclet number as $\tau_{e}=R_{M}^{2}/PeD$. Normalized time is determined from actual time as $t^{\prime }=R_{f}T/\tau_{e}$, while normalized filling time is $t_{Fr}^{\prime }\approx \ln(Pe/2)$. Pe was, therefore, adjusted using known values of $R_{f},\;\;D,\;\mathrm{and}\;R_{M}$ to match the peak at the longest time shown. There is some noise in perimeter regions caused by paper granularity, and some oscillations caused by mechanical changes. Both degrade the ramp profile, but a good match is obtained to a Gaussian distribution near the concentration peak (red line) and to the full profile (green line) for Pe≈500. Similar results were obtained for the minor axis, but with a small increase in Pe. These results confirm the basic principles of inward radial concentration.

## V. COMPARATIVE CONCENTRATION

In this section, we compare linear and radial concentration on patterned substrates. The first difficulty is again measurement and control of the evaporation rate. Concentration was, therefore, measured for strip and wedge shapes on a common substrate, using flow parallel to a common axis, under conditions when the temperature and humidity must be identical. The second is to provide a repeatable geometry. Wax and laser patterning were compared, and similar results were obtained in each case. However, laser patterning gave improved edge definition and was, therefore, used in the results that follow.

### A. Materials

**FIG. 15.**
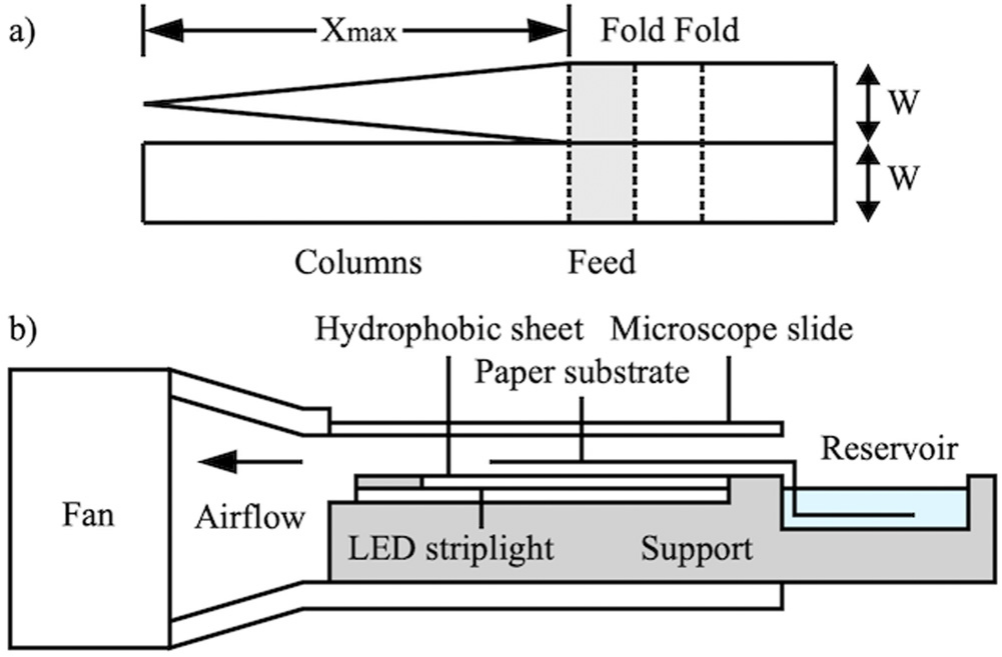
(a) Layout of patterned substrates; (b) arrangement for concentration on strip- and wedge-shaped devices.

Figure 15(a) shows the substrate layout, which contained adjacent parallel and tapered columns of length $X_{max}=30\;\mathrm{mm}$ and width W=8mm. In each case, a short (5mm) non-evaporative feed section linking to a wick inserted into the reservoir was incorporated.

The substrate was 1 Chr paper (3001-845, Whatman, thickness and pore size 0.18mm and 11μm), which has the fast-flow direction parallel to the long axis of the paper. Patterning was carried out with a Lotus Blu 100 CO_2_ laser (Lotus Laser Systems, Basildon, UK). The solute was again Brilliant Blue FCF, at a similar initial concentration.

### B. Apparatus

Figure 15(b) shows a schematic of the equipment used, which consisted of a nylon support designed to fit in a Perspex tube. The support was machined to contain a reservoir and an LED striplight, and the upper surface of the tube was replaced with a microscope slide to improve imaging. The substrate was folded to define cantilever and feed sections and taped to a thin, translucent, hydrophobic polymer sheet (which provided mechanical support, but restricted evaporation to one surface) above the striplight, with the feed linking to the reservoir. The support was then inserted into the tube.

**FIG. 16.**
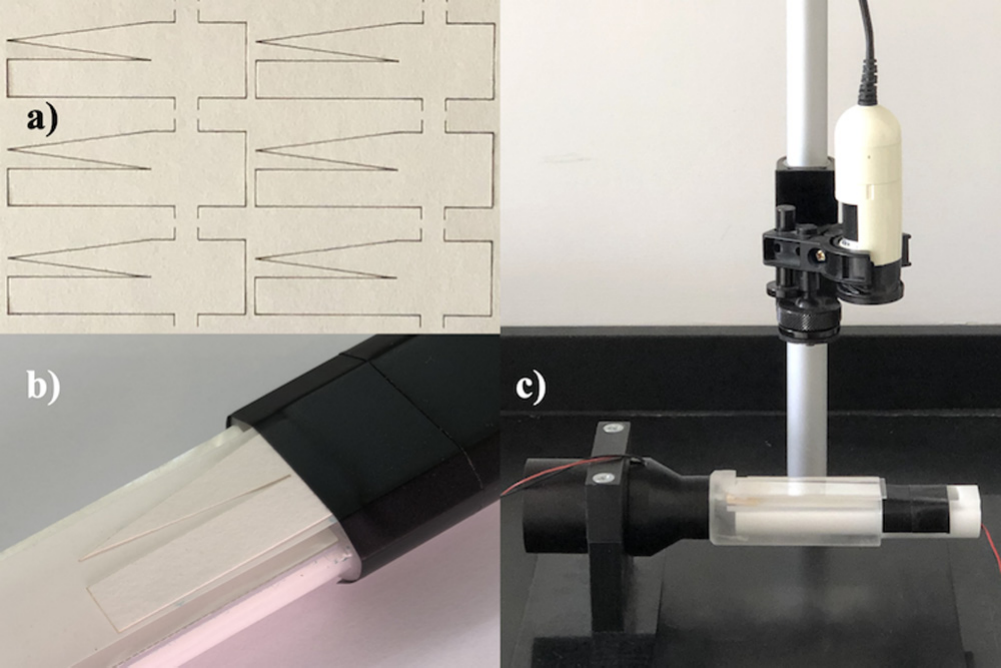
(a) Laser-patterned substrates, (b) substrate on support, and (c) complete rig.

This arrangement provided a rectangular duct with walls ∼2mm from the paper on either side. Airflow was induced using the axial fan, linked to the duct by a tapered adaptor. The substrate was prefilled with water and concentration was monitored by time-lapse photography. Figure 16(a) shows laser-patterned paper devices, Fig. 16(b) shows a substrate mounted for measurement, and Fig. 16(c) shows the complete rig. Images were processed by extracting the brightness along lines through device centers and analyzing results as before.

### C. Results

**FIG. 17.**
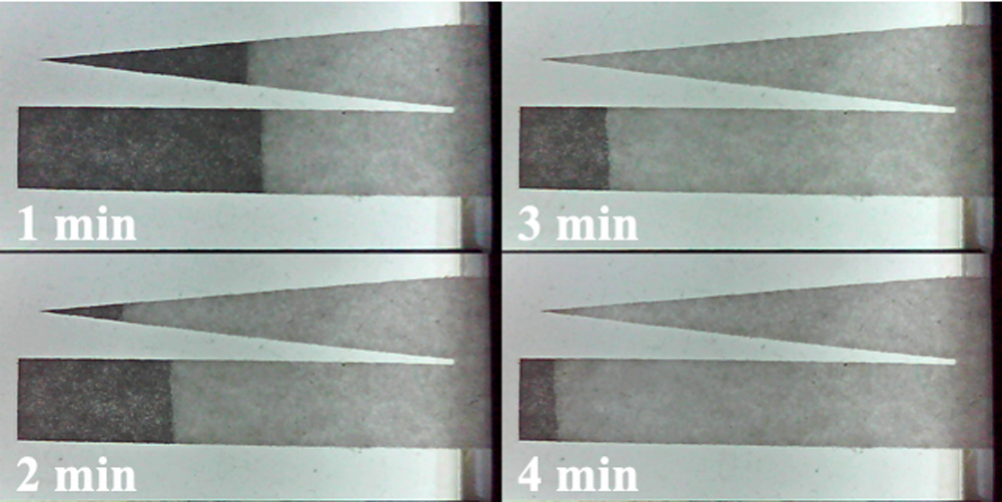
Time-lapse photographs of initial wetting.

Some departures from ideal behavior were again noted. Figure 17 shows photographs during initial wetting, at the times indicated. There is minor evidence of width-dependent flow, but flow profiles across both channels are largely flat.

**FIG. 18.**
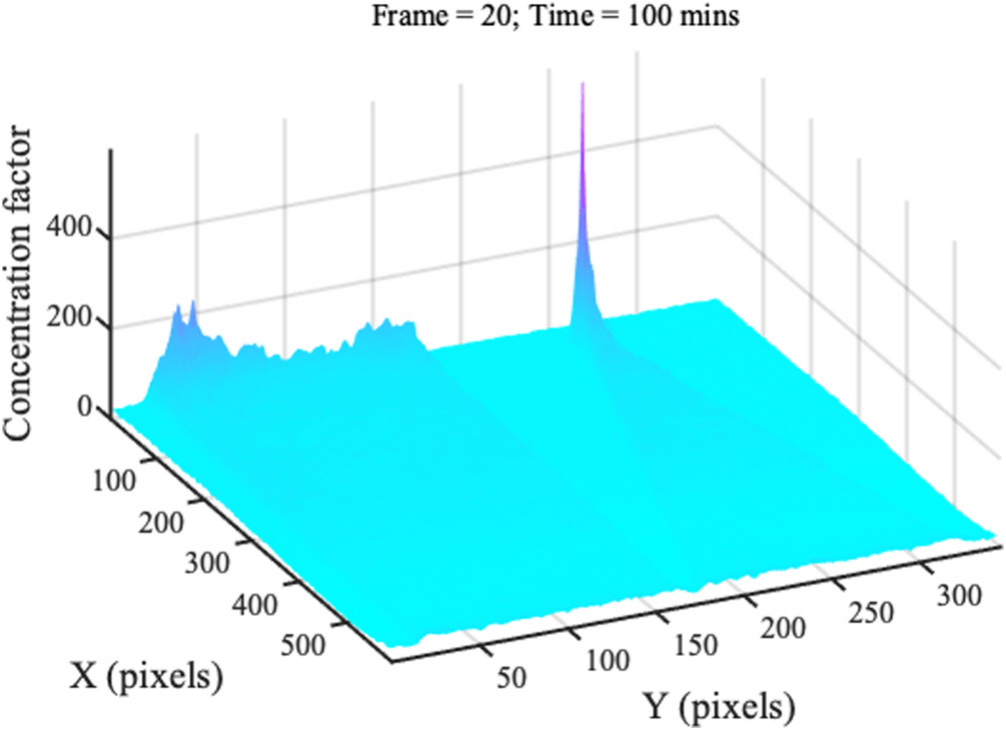
3D presentation of typical concentration profiles.

Similarly, Fig. 18 shows concentration profiles from a typical experiment, plotted in 3D. Here, the strip channel is on the left and the wedge on the right. The wedge concentrates as expected, but there is clear evidence of concentration at the strip corners, which must act as stagnation points for locally radial systems. This effect must reduce 1D concentration rates, but some mitigation was achieved using relatively wide strips.

**FIG. 19.**
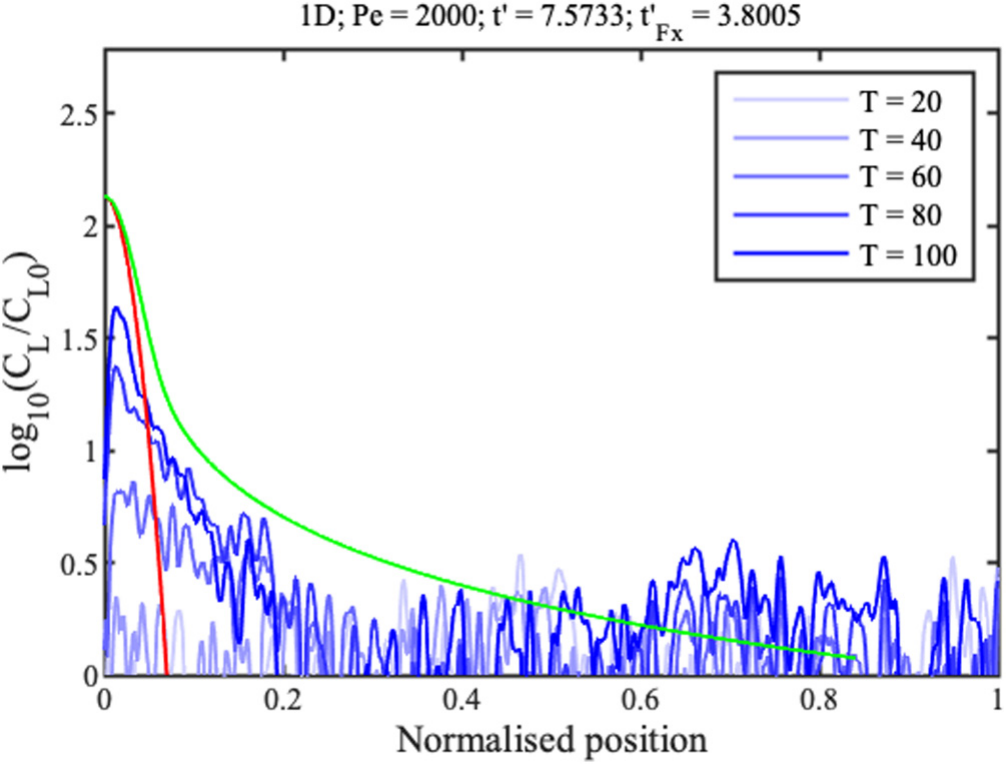
1D concentration profiles. Blue lines show measured data; red and green lines show theoretical fits to the concentration peak and full profile.

**FIG. 20.**
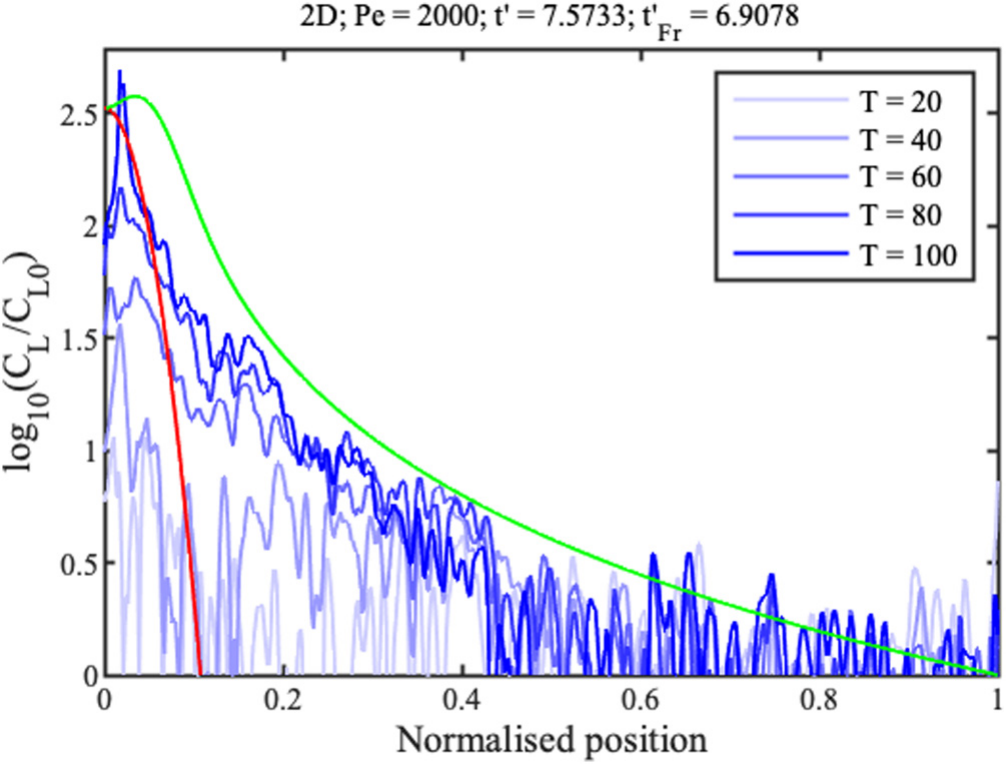
2D concentration profiles. Blue lines show measured data; red and green lines show theoretical fits to the concentration peak and full profile.

The blue lines in Figs. 19 and 20 show the corresponding concentration profiles at the times indicated in minutes up to 1 h 40 min, which confirm an increase in ramp slope and concentration rate for the wedge geometry.

**FIG. 21.**
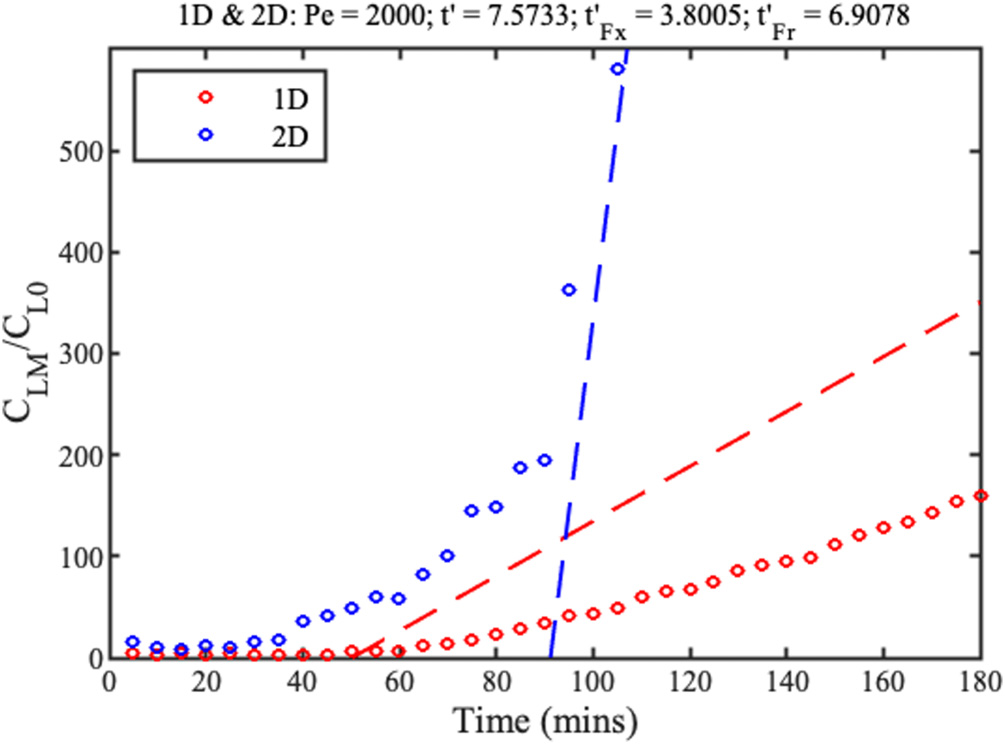
Time variation of peak concentration in 1D and 2D. Points show experimental data; dashed lines show theory.

The points in Fig. 21 compare the time variation of peak concentration in each case. These data confirm the advantage of the radial geometry: although the filling time is longer, the concentration rate after filling is higher, and a peak concentration factor of ∼580 is demonstrated. Similar results were obtained in repeated experiments, with exact details depending on the Péclet number.

Theory was matched to experiment as before, using in addition the 1D filling time $t_{Fx}^{\prime }\approx \ln\left(\sqrt{Pe}\right)$. However, it was difficult to match both sets of data using a single fitting parameter. Instead, Pe was estimated by matching theory to 2D data, and then used to predict 1D results. The red profiles in Figs. 17 and 18 show Gaussian concentration peaks (red lines) and full profiles (green lines) for Pe≈2000. This value is 4x larger than that in Sec. V B, despite the use of single-sided evaporation. Similarly, the dashed lines in Fig. 19 show the approximate linear variations of peak concentration. For the wedge, the experimental concentration profile and rate both agree reasonably with theory. For the strip, experimental results lie below theoretical estimates. This poor performance is ascribed to diversion of concentrate to strip corners. However, the qualitative agreement between theory and experiment is reasonable given the omission of anisotropy and edge effects.

## VI. CONCLUSIONS

Paper-based evaporation concentrators with linear and radial geometry have been compared. Approximate analytic solutions to advection–dispersion equations have been presented for complete concentration profiles using infinite sources. These show that 1D concentration rates scale as √Pe, while 2D rates scale as Pe. Radial concentration, therefore, involves longer initial filling times but then quickly offers faster concentration. Radial concentration has been demonstrated on filter paper fed from an “infinite” perimeter source using optical transmission through dye for quantitation, in a reverse of the “coffee stain” experiment. Under ambient conditions, concentration factors of several 100 were obtained on 60 mm diameter substrates in timescales of 10 h. Airflow-enhanced concentration has been compared in strips and wedges, using a laser-patterned chromatography paper. The faster concentration of wedge geometries has been confirmed and high (>500) concentration factors have been demonstrated on a timescale of 2 h using 30 mm long columns. This advantage is only increased by diversion of concentrate to corners in strip-shaped columns.

Further work is required to describe and exploit this effect. More realistic kinetics must be included in evaporation, flow, and diffusion models, together with anisotropy and boundary effects, and the consequence of saturating and competitive adsorption at high concentrations must be understood. Exact analytic solutions to ADEs with spatially varying, nonlinear coefficients are then required for different initial conditions. Anisotropy could be mitigated using alternative porous media such as thin-layer chromatography substrates. Applications might lie in reconcentration of adsorbed samples using pure solvent (for example, of blood spots in blood analysis devices^62^). Applications for wedge-shaped devices may be as pre-concentrators for tapered spray sources,^63^ and concentration effects may explain the results observed for paper spray with extended solvent supply.^64^

## Data Availability

The data that support the findings of this study are available within the article.
